# Decreased postoperative nausea and vomiting with dexmedetomidine after off-pump coronary artery bypass grafting

**DOI:** 10.1186/cc9771

**Published:** 2011-03-11

**Authors:** H Okawa, T Ono, E Hashiba, T Tsubo, H Ishihara, K Hirota

**Affiliations:** 1 Hirosaki University Graduate School of Medicine, Hirosaki, Japan

## Introduction

Postoperative nausea and vomiting (PONV) is one of the factors that affect the quality of postoperative patient care. We would like to report possible antiemetic effects of dexmedetomidine (DEX), a selective α_2_-agonist sedative, in patients after off-pump coronary artery bypass grafting (OPCAB).

## Methods

Local Research Ethics Committee approval and written informed consent from patients or next of kin were obtained before this study. Patients after OPCAB were allocated into two groups (sedated with DEX; DEX(+), *n *= 123 and no sedation; DEX(-), *n *= 134). The incidence of PONV, postoperative morphine consumptions and amount of gastric fluid drained via a nasogastric tube were compared in the two groups.

## Results

There were no significant differences in the patients' profiles and intraoperative opioid consumptions. Eight patients in the DEX(+) group had PONV whereas 35 patients had PONV in the DEX(-) group (6.5% vs. 26.1%, *P *< 0.01) during a postoperative observation period of 12.5 (3.2) and 11.8 (2.4) hours, respectively (mean (SD)). The ratio of patients who required morphine for postoperative pain relief was lower in the DEX(+) group than the DEX(-) group (67.5% vs. 83.6%, *P *< 0.01), presumably due to analgesic effects of DEX. Analysis of individual patients revealed that five out of eight patients and 12 out of 35 patients had PONV after morphine use in the DEX(+) and DEX(-) groups, respectively. Repeated analysis without those patients revealed the same tendency (3.4% vs. 18.2% had PONV in the DEX(+) and DEX(-) groups, respectively; *P *< 0.01) as obtained in all the patients, suggesting antiemetic effects of DEX *per se*. There were no significant differences in the amount of gastric fluid drained via a nasogastric tube between groups.

## Conclusions

DEX is reported to inhibit gastrointestinal transit and gastric emptying like morphine. According to this report, the decreased incidence of PONV in the DEX(+) group in our study is not likely to be caused by peripheral effects of DEX on the gastrointestinal tract. It is widely recognized that morphine induces PONV, and we analyzed the incidence of PONV without patients who had any suspicion of morphine-induced PONV, obtaining the same result. According to these considerations, we would like to conclude that DEX could have antiemetic effects *per se*.
